# Efficient Multi-Object Detection and Smart Navigation Using Artificial Intelligence for Visually Impaired People

**DOI:** 10.3390/e22090941

**Published:** 2020-08-27

**Authors:** Rakesh Chandra Joshi, Saumya Yadav, Malay Kishore Dutta, Carlos M. Travieso-Gonzalez

**Affiliations:** 1 Centre for Advanced Studies, Dr. A.P.J. Abdul Kalam Technical University, Lucknow 226031, India; rakeshchandraindia@gmail.com (R.C.J.); saumyay.15@gmail.com (S.Y.); 2 Institute for Technological Development and Innovation in Communications (IDeTIC), University of Las Palmas de Gran Canaria (ULPGC), 35017 Las Palmas de G.C., Spain; carlos.travieso@ulpgc.es

**Keywords:** artificial intelligence, assistive systems, computer vision, deep learning, machine learning, object recognition, visually impaired person, YOLO-v3

## Abstract

Visually impaired people face numerous difficulties in their daily life, and technological interventions may assist them to meet these challenges. This paper proposes an artificial intelligence-based fully automatic assistive technology to recognize different objects, and auditory inputs are provided to the user in real time, which gives better understanding to the visually impaired person about their surroundings. A deep-learning model is trained with multiple images of objects that are highly relevant to the visually impaired person. Training images are augmented and manually annotated to bring more robustness to the trained model. In addition to computer vision-based techniques for object recognition, a distance-measuring sensor is integrated to make the device more comprehensive by recognizing obstacles while navigating from one place to another. The auditory information that is conveyed to the user after scene segmentation and obstacle identification is optimized to obtain more information in less time for faster processing of video frames. The average accuracy of this proposed method is 95.19% and 99.69% for object detection and recognition, respectively. The time complexity is low, allowing a user to perceive the surrounding scene in real time.

## 1. Introduction

Vision impairment is one of the major health problems in the world. Vision impairment or vision loss reduces seeing or perceiving ability, which cannot be cured through wearing glasses. Navigation becomes more difficult around places other than the visually impaired person’s own home or places that are not familiar. Vision impairment is classified into near and distance vision impairment. In near vision impairment, vision is poorer than M.08 or N6, even after correction. Distance vision impairment is classified into mild, moderate, severe, and blindness based on visual acuteness, when it is worse than 6/12, 6/18, 6/60, and 3/60, respectively [1]. About 80% of people who suffer from visual impairment or blindness belong to middle- and low-income countries, where they cannot afford costly assistive devices. The problem arises due to an increase in age or population [2]. Vision impairment can be due to many reasons such as uncorrected refractive errors, age-related eye problems, glaucoma, cataracts, diabetic retinopathy, trachoma, corneal opacity, or unaddressed presbyopia [3].

Apart from medical treatment, people use various aids for rehabilitation, education, social inclusion, or work. A white cane is used by visually impaired people around the world. The length of the cane is directly proportional to the range of touch sensation or the detection of obstacles. Guide dogs are also used as walking assistance, where the dog makes the user aware of obstacles or for stepping up and down. However, guide dogs are unable to give directions in complex cases. People also make use of GPS-equipped assistive devices, which help with navigation and orientation for a particular position. These kinds of devices are accurate in terms of location, but are ineffective in case of obstacle avoidance and object identification. Echolocation [4] is another technique used by blind people in which echoes of sounds made by simple mouth clicks are used to detect silent objects in front of them.

Braille helps a visually impaired or blind person to obtain information, but it is limited to people who have knowledge of it. Information in braille characters can be installed in most places, but it is not practical to install it everywhere and convey full information. Currency notes also feature the tactile marks with raised dots to allow the person to identify the banknote. However, these tactile marks vanish after some time, and then it is not easy for a blind person to differentiate between banknotes. Refreshable braille displays, screen magnifiers, and screen readers are also used to obtain information while using computer or mobile systems.

Visually impaired people use Electronic Travel Aids (ETA) to detect obstacles and identify services to provide safe and informative navigation. A hardware-based robotic cane is proposed for assistance in walking. It has an omnidirectional wheel assisted with a high-speed processing controller using a LAM-based linearization system with a non-linear disturbance observer. It maintains the balance of the person and reduces the risk of falling. The length, cost, and weight are other parameters, which can be optimized for better support [5]. An Electronic Mobility Cane (EMC) is designed for vision rehabilitation of visually impaired people to provide assistance and detect obstacles [6], where a logical map is constructed to obtain information about the surrounding environment. Output information is conveyed in the form of audio, vibration, or voice. A haptic device such as a short cane with smart sensors is proposed in [7] to provide information about obstacles. Different ultrasonic sensors are associated with providing the same stimuli of a traditional stick without touching obstacles. Another multi-sensor ETA device proposed in [8] has a pair of eyeglasses that guide people in a safe and efficient manner using ultrasonic and depth sensors to provide a navigational guide to the visually impaired person. A cane robot is proposed in [9] for assistance and fall prevention. Some object-recognition techniques are based on Quick Response (QR) codes and barcodes to identify different types of objects at various places such as in shopping malls, etc. [10,11], which requires an advanced infrastructure.

A smart cane and obstacle-detection system for visually impaired people with multiple sensors has been designed with model-based state-feedback control [12]. A linear–quadratic regulator (LQR) based controller is also integrated for the optimization of an actuator’s control actions along with position tracking. A white-cane system that is composed of IC tags is designed in [13], which supports independent walking of visually impaired people in indoor space. Colored lines on the floor for navigation is sensed by the cane, and information to reach the destination is given with vibrations and voice prompts. An intelligent system is proposed in [14] which contains map information for independent navigation walking for visually impaired people while walking in indoor space. The color on the floor is recognized through the one-chip microprocessor and Radio Frequency Identification (RFID) system. Tactile zooming is discussed in [15] for graphics, which make it easy for visually impaired people to obtain information by magnification. Navigation assistance for the visually impaired (NAVI) is developed to assist through sound commands using an RGB-D camera [16]. Automatic recognition of clothing patterns is done in [17] for visually impaired people, which can identify 11 colors of cloth. The system consists of a mounted camera on goggles, a microphone, a Bluetooth ear piece, and a computer. A wearable device is designed using a haptic strap, sensor belt, and vibration motors to detect different types of obstacles and allow for safe navigation [18]. An object-detection method is proposed in [19], which is based on a deformable grid (DG), which depends upon the motion of an object and can detect the risk of collision.

In [20], an automatic quantization algorithm is developed for deep convolutional neural network (DCNN)-based object detection that uses a smaller number of bits and reduces the hardware cost compared to traditional methods. The main challenge in developing an assistive framework using a CNN architecture is to increase the accuracy for the classification task while maintaining an acceptable computational workload [21]. A DEEP-SEE FACE framework-based assistive device is introduced for improving the cognition and communication of visually impaired people by recognizing known faces and differentiating them from unknown faces [22]. A light detection and ranging (LIDAR)-assisted system is proposed in [23] to obtain spatial information through the stereo sound of different pitches. A tracking system for indoor and outdoor navigation using computer vision algorithms and dead reckoning to help visually impaired people has been implemented in a smartphone [24]. An electronic device for the automatic navigational assistance of a visually impaired person, named NavCane, is developed in [25] for the obstacle detection of various types with different types of sensors at different positions of a white cane. SUGAMAN [26] is a framework developed to describe floor plans using proximity-based grammar and learning annotation. It utilizes the text information in floor plan images to develop proper navigation and obstacle avoidance for visually impaired persons. A wearable deep learning-based drug pill recognition system for the improvement in safety to visually impaired people using medicines works by reducing the risk of taking incorrect drugs [27]. An assistive device to help visually impaired people using partial visual information and machine learning techniques which enables semantic categorization for the classification of obstacles in front of them achieves a highest accuracy of 90.2% [28].

Science and engineering can make technical interventions to the lives of visually impaired people in making them independent to navigate and perceive objects around them. Many devices have been proposed to assist a visually challenged person, but most of the devices focus either on object detection through computer vision or on obstacle detection through different sensors, such as GPS, distance sensors, etc. However, the effective utilization of sensor-based technologies and computer vision could result in a highly efficient and supportive device, to make them aware of the surroundings.

The main contribution of the proposed work is to design an artificial intelligent fully automated assistive technique for visually impaired people to perceive the objects in the surrounding and provide obstacle-aware navigation, where auditory inputs are given to users in real-time. Images of objects that are highly relevant in the lives of the visually challenged are trained using deep learning neural networks. Augmentation and manual annotation are performed on the dataset to make the system robust and free from overfitting. Both sensors and computer-vision based techniques are integrated to provide convenient a travel-aid to visually impaired people, through which a person can perceive multiple objects, detect obstacles and avoid collisions.

The whole framework is standalone and designed for low-cost processors, so that the visually impaired can use it properly without internet connectivity. The detected output is also optimized to ensure faster frame processing and a greater extraction of information—i.e., count of objects—in a shorter time period. The proposed system can easily differentiate between obstacles and known objects. The proposed methodology can make a significant contribution to assist visually impaired people compared to previously developed methods, which were only focused on obstacle detection and location tracking with the help of basic sensors without use of deep learning. With the use of the proposed methodology, users can interpret more about the things going around and receive obstacle-aware navigation as well.

The rest of the paper is organized as follows: First, the methodology is explored in Section 2, followed by the experimental results presented in Section 3. Finally, our conclusions and ideas for future work are discussed in Section 4.

## 2. Methodology

**Figure 1:**
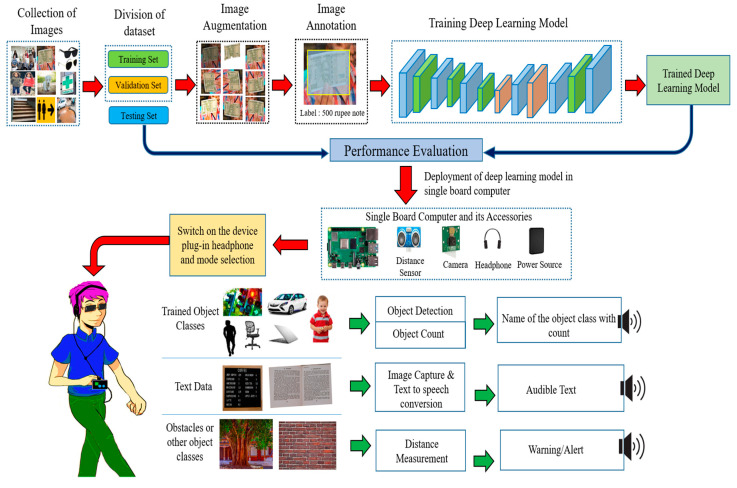
Block Diagram for proposed methodology.

In this section, the whole process is explained to provide navigation assistance to visually impaired people, which consists of the preparation and pre-processing of the dataset, augmentation, annotation and dataset training on the deep-learning model. The block diagram for proposed methodology is represented in Figure 1.

### 2.1. Dataset for Visual Impaired People

Many datasets are available for object detection, such as PASCAL [29], CIFAR 10 [30], IMAGENET [31], SUN [32] and MS COCO [33] but these contain limited classes from the perspective of assisting visually impaired persons. Thus, there is a need to add more objects in existing datasets so that they can help visually disabled persons to be socially independent. A survey was conducted in visually disabled schools and colleges to select more relevant objects to train a deep-learning model. The dataset was generated from multiple sources and devices, in different sizes and pixels. Various lighting conditions and capturing angles were used to make more variations in the collected dataset. The banknote/currency notes were also included in the dataset, to perform cash transactions with ease. Thereafter, those images which had less than 10% area of the targeted object or any deformities—such as flickering, blur or noise to more than an acceptable extent—were eliminated. After that, augmentation variants were applied to the captured and collected images.

### 2.2. Image Augmentation

**Figure 2:**
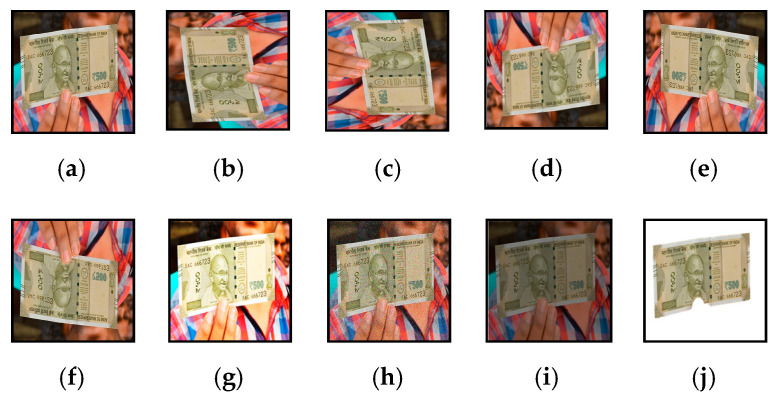
Different image augmentation techniques on acquired images: (**a**) Original Image; (**b**) 90° left rotation; (**c**) 90° right rotation; (**d**) 180° Horizontal rotation; (**e**) Horizontal Flip; (**f**) Vertical Flip; (**g**) Increased Brightness; (**h**) Addition of noise; (**i**) Low contrast; (**j**) Background Removal.

All collected images were then augmented to resist the trained model from overfitting and to perform more robust and accurate object detection for visually impaired persons. Various augmentation techniques, such as rotation at different angles, skewing, mirroring, flipping, brightness levels, noise levels, and a combination of these techniques, was used to enrich the dataset to many folds, shown in Figure 2. As banknotes are also a part of daily life, various images of different denominations of banknotes were collected and augmented before training the neural network to recognize banknotes efficiently and accurately.

### 2.3. Image Annotation

All images were annotated manually with the LabelImg tool and the bounding box was made around the object without taking extra unnecessary areas. The information about the images, such as the size of the image, size, and position of the bounding box or bounding boxes (in case of multiple instances or multiple objects in the same image), were recorded and saved into the “.xml” format. Once the images were annotated, the respective annotation files were also generated. The final dataset, that consists of annotated images and respective annotation files, was divided into two sets—training and validation. Then, the YOLO-v3 model is trained with the generated dataset either through transfer learning or with direct training.

The transfer learning method requires a pre-trained model and it will be beneficial when a similar dataset is already trained over this model and respective generated trained model files will be used for transfer learning. Due to this, weight adjustment takes less time compared to the case when training the dataset for the first time. As weight adjustment and loss in each convolving layer reduce in a shorter time, the transfer learning method can also be used to retrain the dataset when the training got abrupt due to any reasons.

### 2.4. Dataset Training on Deep-Learning Model

In the YOLO-based object detection [34], the given image was divided into grids of S × S where S = a number of grid cells in each of the axes. There, each unit of the grid was accountable to detect the targets which were getting into it. Then, a corresponding confidence score was predicted for the B number of bounding boxes by each of the grid units. The confidence score represents the similarity with the desired object and maximum likelihood represents a higher confidence score of the corresponding object. In other words, it defines the presence and absence of any object class in the image. In the same way, if the object did not contain the desired object, the confidence score would be zero. If the object was contained by the predicted bounding box, then the confidence score would be calculated by the interaction in between both bounding boxes, i.e., predicted and ground truth represented by the Interaction over Union (IOU). Equation (1) is used to calculate the confidence score in the given input image.

(1) $$CS\;=\;P_{r}\left(Obj\right)*IOU_{Groundtruth}^{Predicted}$$

where, *CS* = Confidence Score, $P_{r}\left(Obj\right)$ represents the probability of the object and $IOU_{Groundtruth}^{Predicted}$ represents the IOU of predicted and ground truth bounding boxes.

Loss function for YOLO architecture is given by Equation (2).

(2) $$\begin{array}{ll} \mathrm{Loss}=\lambda_{coord} & \overset{S^{2}}{\underset{i=0}{\sum }}\overset{B}{\underset{j=0}{\sum }}{𝟙}_{ij}^{obj}\left[{\left(x_{i}-{\hat{x}}_{i}\right)}^{2}+{\left(y_{i}-{\hat{y}}_{i}\right)}^{2}\right]\\ & +\lambda_{coord}\overset{S^{2}}{\underset{i=0}{\sum }}\overset{B}{\underset{j=0}{\sum }}{𝟙}_{ij}^{obj}\left[{\left(\sqrt{w_{i}}-\sqrt{{\hat{w}}_{i}}\right)}^{2}+{\left(\sqrt{h_{i}}-\sqrt{{\hat{h}}_{i}}\right)}^{2}\right]\\ & +\overset{S^{2}}{\underset{i=0}{\sum }}\overset{B}{\underset{j=0}{\sum }}{𝟙}_{ij}^{obj}{\left(C_{i}-{\hat{C}}_{i}\right)}^{2}\\ & +\lambda_{noobj}\overset{S^{2}}{\underset{i=0}{\sum }}\overset{B}{\underset{j=0}{\sum }}{𝟙}_{ij}^{noobj}{\left(C_{i}-{\hat{C}}_{i}\right)}^{2}+\overset{S^{2}}{\underset{i=0}{\sum }}{𝟙}_{i}^{obj}\underset{c\in classes}{\sum }{\left(p_{i}\left(C\right)-{\hat{p}}_{i}\left(c\right)\right)}^{2} \end{array}$$

where, ${𝟙}_{ij}^{obj}$ denotes the *j^th^* bounding box predictor in the *i*^th^ cell, which is also responsible for prediction, and ${𝟙}_{i}^{obj}$ denotes if the object appears in cell *i*.

YOLO-v3 [35] is an upgraded version of YOLO and YOLOv2 [36] for object detection in real-time and accurately. YOLO-v3 uses logistic regression is utilized instead of Softmax to predict the objectness score for each bounding box. Thus, multi-label classification and class prediction can be performed using YOLO-v3. Feature Pyramid Networks (FPN) in YOLO-v3 makes three predictions for each location of the input frame and features are extracted from each prediction, which include boundary box and objectness scores.

**Figure 3:**
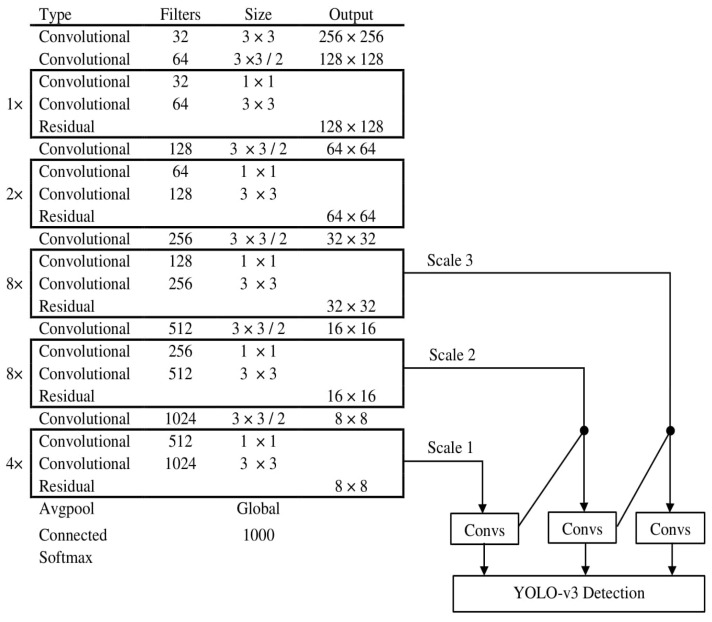
Network Structure of YOLO-v3.

Darknet-53 is used as a feature extractor in YOLO-v3 that is composed of 53 convolutional layers. It runs at the highest measured floating-point operation speed, which indicates that the network is more successful when applying GPU resources [37]. The network architecture of YOLO-v3 with Darknet-53 is shown in Figure 3.

In the training neural network, the predictions were made through the following Equations (3)–(6):

*b_x_ = σ(t_x_) + c_x__⋯_* (3)

*b_y_ = σ(t_y_) + c_y_* (4)

*b_w_ = p_w_ e^tw^* (5)

**Figure 4:**
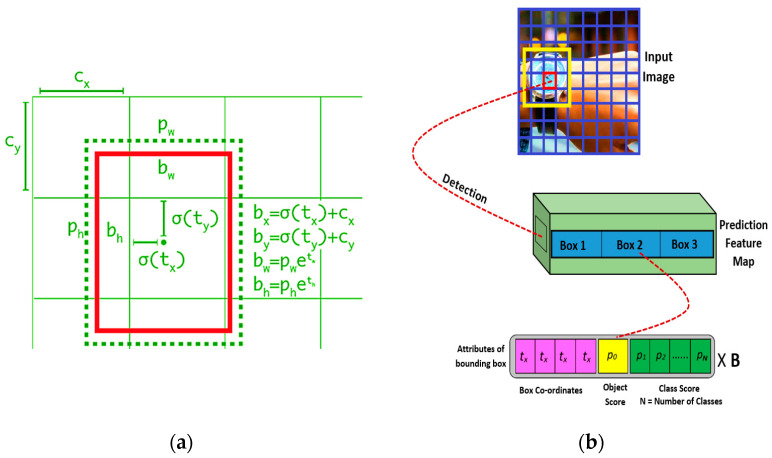
(**a**) Bounding Box Prediction; (**b**) Object detection with YOLO-v3.

*b_h_ = p_h_ e^th^* (6)

where (*t_x_, t_y_, t_w_, t_h_*) are four coordinates that were predicted for each of the bounding boxes. (*c_x_, c_y_*) is the cell offset from the top left image corner, and (*p_w_, p_h_*) are the width and height of the bounding box prior. The diagrams for bounding box prediction and object detection with the training model are shown in Figure 4.

Once the CNN was trained with the dataset, the final trained model was equipped in the object detection framework. A live video feed was associated to the framework and image frames were subsequently captured. Captured frames were pre-processed and fed into the trained model, and if any object which was trained with the model was detected, a bounding box was drawn around that object and a respective label was generated for that object. Once all objects were detected, the text label was converted into speech, or a respective audio label recording was played, and subsequently, the next frame was processed. The Algorithm 1 elaborates the steps of object detection for a visually impaired person after the training of the dataset is as follows:
**Algorithm 1.** Object detection for visually impaired person after training of dataset.*Input: Captured image from the Camera* *Output: Audio for the label of the detected object* **Step 1:***Save the captured image, I* **Step 2:***Pre-processing of the image*    Resize the image in dimensions w*h     *where, w = number of pixels in the x-axis*      *h = number of pixels in the y-axis*     *Increase the contrast value for I* **Step 3:***Load the trained deep-learning model and its parameters* **Step 4:***Image I is processed with the deep-learning model*    *detections = detectObjectsFromImage(input_image = I)* **Step 5:***Save processed output image, O*      **for***detections*        *Bounding Box prediction (b_x,_ b_y,_ b_w,_ b_h_)*        *Percentage probability of the object*        *Label, l = name of the detected object*        *Text to speech conversion for l*      **end**


The proposed module consists of a DSP processor with a distance sensor, camera, and power supply. Speakers or headphones are associated with the DSP processor to perceive predictions as an audio prompt.

Output information optimization was further performed to increase the robustness of the system. If an object is detected in the captured image frame, equivalent audio is played after the detection of an object to convey information to the user. Thus, the information transmission time will increase with an increase in the number of the objects in current image frame and cause a delay to processing the next frame. This problem is not discussed in many research articles where such work is conducted. Frame processing time in blind assistive devices is different for a normal human and visually impaired persons. In the case of assistance for visually impaired people, the frame processing time also includes the time necessary to convey detection information as audio or vibrations. Thus, even though the machine learning model processes the frames in real-time, it takes a lot of time to process the next frame, as it has a dependency on the number of the objects present in the current frame and the length of the name of object. For example, the time taken to pronounce “car” is less than that required to pronounce “fire extinguisher”. Thus, three steps have been taken to deal with these kinds of problems. First, all audio files for the name of objects label are optimized such that there is no silence in recording, except the space in between two words. Recording playback speed is increased to the extent that it still sounds clear and understandable.

Second is the case where the same kind of objects exist multiple times in the captured frame. For example, in a case where 5 people are present in the scene, the conventional system will take the equivalent of five times to prompt the word “person”, or more (because of a time gap in between pronouncing two words). To optimize this, the object counter is added with a trained model that counts the number of objects of same category in current image frame, processes it, and conveys a piece of audio information with both “number of objects” and “name/label of an object”. Thus, the time taken to prompt “person” five times is reduced to “5 person”. Consequently, the time taken to process the next frame is reduced, which results in a smaller instant of time to convey information.

**Figure 5:**
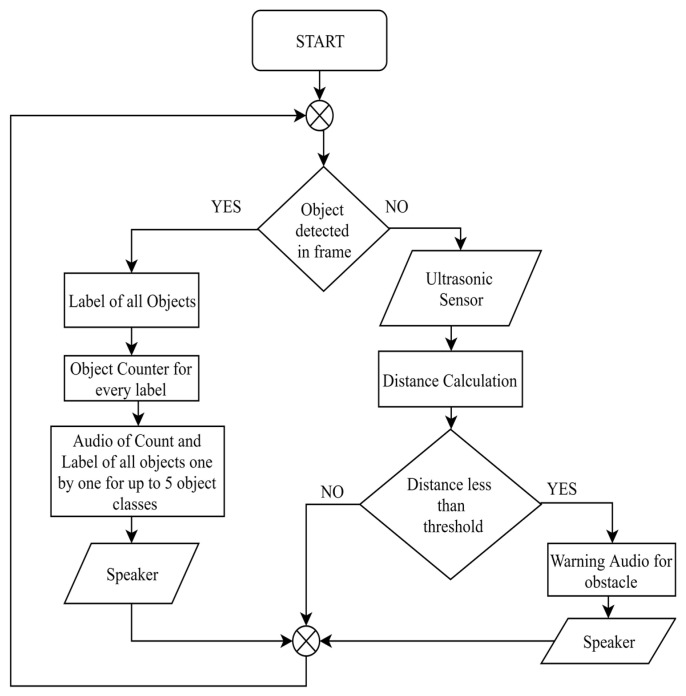
Information Optimization and Object–Obstacle Differentiation.

Third is the case in which a number of multiple objects of various categories are present in the captured scene, which can require a considerably longer time to convey audio information to the user. To deal with this issue, the number of object categories is limited to three, but can be extended to five object classes for indoor circumstances. This means that even though the number of objects which were detected is higher, the system will convey the information of all objects of only the first three categories and then process the next frame. With these three improvements in information transmission, the processing time between two frames is reduced. A flow chart diagram for the optimized information transmission with the object counter is shown in Figure 5.

If none of the trained objects are detected in the captured frame, then it will calculate the distance through the ultrasonic sensors. If the calculated distance is less than the threshold, then it will be considered as an obstacle. Otherwise, if the calculated distance is more than the threshold, then the next image frame will be captured and processed.

All previous inventions and research works for blind or visually impaired people which use ultrasonic sensors to detect obstacles define their range and play a warning sound whenever an obstacle comes across the sensor. When the calculated distance through the ultrasonic sensor is below the threshold value, the device makes an acoustic warning or vibrates, but it can be irritating for a visually impaired person who is standing in a crowd and repeatedly listening same prompt or continuous vibrations. So, one of the objectives of the proposed system is to differentiate between trained objects and obstacles.

The system first analyses the current frame for object detection—if an object is detected which means the object is in front of the device, then there is no need for searching another obstacle. If no objects are identified in present frame, then it takes input from an ultrasonic sensor regarding the distance from the object, and if the calculated distance is less than the threshold, then it treats object as an obstacle and warns the person through an auditory message, as shown in flow chart in Figure 5. A vibration motor can also be associated so that it can vibrate at that instance. However, an auditory response is good in many respects, as it does not annoy a person unlike vibrations, and the power requirement is less compared to a vibration motor.

**Figure 6:**
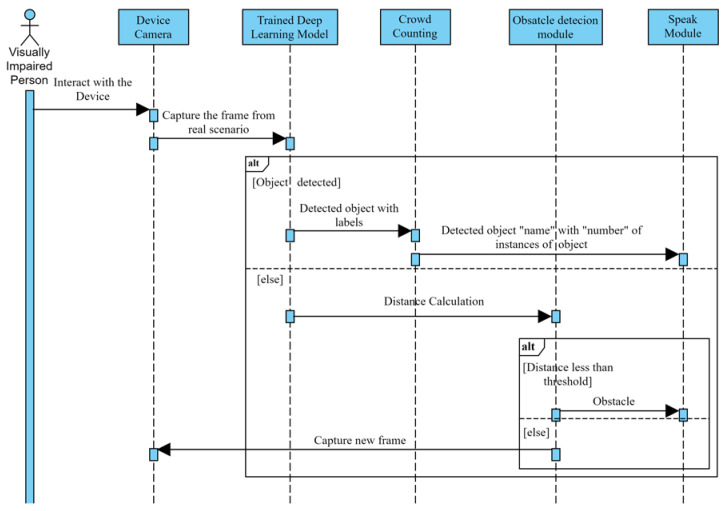
Activity Diagram for the proposed assistive approach for visually impaired people.

Different modes are designed in the device to provide wider assistance such as indoor, outdoor or text-reader mode. The activity diagram for the working of the assistive framework is illustrated in Figure 6.

The indoor mode has a smaller threshold value for the distance of obstacle compared to the outdoor mode. Outdoor mode also has an image enlarge function, so that far objects can be detected early. For example, a car at far distance can be easily detected when enlarged, because after enlarging the image, the number of pixels is increased, and it becomes an easy task to detect that car. This feature provides an audio prompt when the object is at distance, and helps user to be alert to their surroundings, as early detection is crucial in case the user is outside and especially in those scenarios such as when a car is coming towards the person. The text reader mode can be used efficiently where the user has a necessity to read, such as when reading a book, a restaurant menu, etc. To read text, Optical Character Recognizer (OCR) is used after preprocessing the input image frame. Face recognizer can also be associated with the device, where users can identify known persons and family members, which will help in them to be social and secure.

## 3. Experiments and Results

The hardware specifications of training device are i-9 processor, NVIDIA Tesla K80 GPU, having 2496 CUDA cores, 12 GB GDDR5 VRAM. The system is made for hundred objects of different classes. The model is also trained to perform banknote detection and recognition to help in daily business transaction-related activities along with other object detection and navigation assistance for visually impaired people. The whole set-up is implemented in the single board DSP processor and has specifications of 64-bit, quad-core, and 1.5 GHz, as well as 4 GB SDRAM. The 8-megapixel camera used can capture images of 3280 × 2464 pixels with a fixed focus lens.

In total, 650 images of each class were collected and, out of those, 150 images were kept separated for the testing set. The remaining 500 images from each training class were divided into a ratio of 7:3 for training and validation set, respectively. After completing augmentation, the dataset in the training and validation set was increased by 10 times the initial set of images, which resulted in a wide variety of images. The number of images in the given dataset is given in Table 1. Augmentation induces the robustness in the training model. The Deep Learning model is trained with the dataset at an initial learning rate of 10^−3^. Training is performed until the loss is reduced and becomes saturated at a certain epoch. In between the training processes, the trained model files for lower loss can be used to test the detection and recognition performance of the system to conduct a subsequent analysis of the trained system. If a trained model performs poorly with lower loss model files, either the dataset should be increased, or various augmentations should be performed on an existing dataset.

The model file after training on different object classes is tested on a real-time live video feed along with images left for the testing dataset. Table 2 is prepared for the analysis of object detection and recognition accuracy of proposed system. An average accuracy of 95.19% is achieved for object detection and the average recognition accuracy is 99.69%. The results signify that once the object is detected it will be classified properly among the list of object classes, which were trained on a prepared dataset. As objects are trained regressively, the high threshold will also withstand with the accuracy.

**Figure 7:**
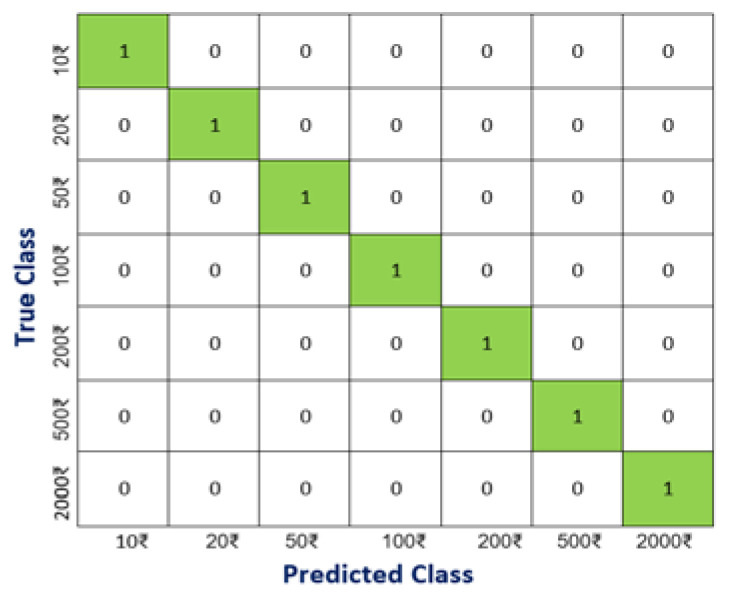
Confusion Matrix.

Confusion matrix is another parameter that can be utilized to check the performance of object detection and recognition on a set of test data whose true values are known. It checks whether the system is capable of differentiating between the two classes of objects after the detection. The higher values in the respective classes show the high differentiation between the two classes. As the similarity in banknotes is greater, confusion matrix for currency notes is shown in Figure 7, taking the highest percentage prediction into consideration. Differentiation between two classes is tougher when two classes are almost similar in appearance. For example, if a banknote of INR 2000 is tested in a folded position and digits are focused, then there could be confusion between INR 20, 200 or 2000. In such cases, the model trained using the dataset predicts the banknote denomination for the captured picture, but it will give a higher value of detection percentage to true value of banknote as it is also trained with the texture of notes. Thus, the overall resemblance with the true value of banknotes will be higher, which can be concluded from the confusion matrix.

The confusion matrix is prepared for a threshold of 0.5; because of this, if the captured image is not proper, there may be chances that image shows some similarity with other banknotes along with the actual currency note. This issue can be easily eliminated by increasing the threshold value or by considering only the highest label prediction probability. Thus, if there is a currency detection mode in the device, that mode must have a higher object detection threshold value than other modes to avoid such ambiguity.

**Figure 8:**
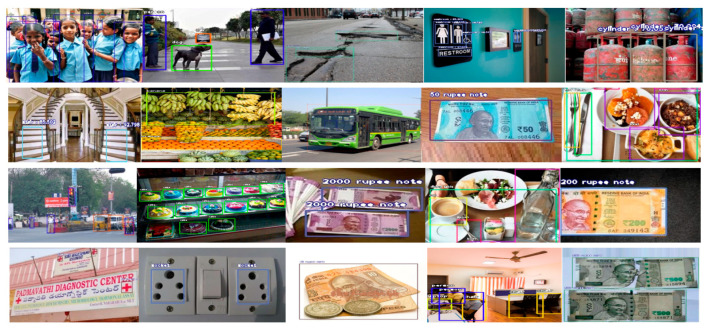
Few results after object detection and recognition.

Once the performance testing is complete, the trained model is loaded onto a small DSP processor and equipped with ultrasonic sensors to detect the obstacles. Results for different object classes in different scenarios are shown below in Figure 8. Trained deep-learning models can detect and recognize the object correctly, which proves the accuracy and robustness of the proposed system.

Different approaches for object classification and object detection were also tested in given datasets, such as VGG-16, VGG-19 and Alexnet. The testing accuracy and processing time for a single image frame are given in Table 3.

Information optimization is performed to get more information in a shorter time duration. The time-domain analysis of the proposed system is given in Table 4 and Table 5. Table 4 explains the parameters and average time taken to perform each step, whereas Table 5 explains object detection in different scenarios, such as single object single instances, single object multiple instances, multiple object single instances, and multiple object multiple instances. All the time parameters are given for a single board DSP processor without GPU support.

Resources are used in an optimized way to reduce energy consumption. Ultrasonic sensors derive power only when the objects are not present in a captured scene. As the model is trained with most of those objects that it comes across in daily life, there is a smaller probability that the ultrasonic sensor will be used, apart from a case where the user is within a closed space with a distance less than the threshold.

The device is programmed to work in a fully automatic manner to perform object recognition and obstacle detection. For switching in between different modes, a person must swipe their hand in front of the device, which can be sensed by ultrasonic sensors to perform mode-switching. Device instructions can also be made multi-lingual by just recording the instructions in other languages. As it does not depend on a computer language interpreter, instructions can also be made for local dialect or language for which proper recordings are not yet available. The device can work in real-time scenarios, as the processing time for object detection is a few milliseconds. The higher the processor, the greater the number of frames per seconds that can be processed.

If a user wants to record image frames, which came across the device, it can be stored in subsequent frames. These frames can also help to construct a proper dataset and to approach the challenging scenario, which can be dealt with to develop much more robust devices. Above all, the whole system is standalone and needs no internet connection to perform object detection and safe navigation.

After training the collected dataset with various image augmentation techniques and multi-scale detection functionality of trained deep neural network, the proposed framework is able to detect objects in different scenarios, such as low illumination, different viewing angles, and various scale objects. The proposed system can work universally in the existing infrastructure which has been used before by visually impaired people.

Proposed work is also compared with the other works in the domain of assistance for the visually impaired and is shown in Table 6.

The performance of the proposed device has been tested on 36 people, including 20 visually impaired and 16 blind-folded people belonging to different age groups. The test is conducted for both indoor and outdoor environments. All were given sticks and a supporting person while using the proposed framework. Various rehabilitation workers and teachers working in this field were also involved to help to conduct the experiments smoothly. Before the trials, all those involved were briefly informed about the device so that users were aware about the experimentation steps. Different obstacles were used in an indoor environment, such as a chair, stairs, humans, walls, etc. While in outdoor environments, trained objects such as cars, humans, and vehicles were used. The visually impaired people previously used blind sticks, which give alerts for objects coming in front of the stick by means of vibrations. It takes a lot of mental effort and attention when walking only with the help of a stick. They experienced lots of problems while using the stick in crowed areas. After using this device, the proposed framework was found to be comfortable and easy to use in crowded areas. The developed technology is found to be highly useful, with which users can also understand the surrounding scenario easily while navigating without putting in too much effort. The proposed aid for visually impaired seems good in the sense that it does not need any prior knowledge about the position, shape and size of object and obstacles.

## 4. Conclusions and Future Scope

An assistive system is proposed for visually impaired persons through which they can perceive their surroundings and objects in real-time and navigate independently. Deep learning-based object detection, in assistance with various distance sensors, is used to make the user aware of obstacles, to provide safe navigation where all information is provided to the user in the form of audio. A list of highly relevant objects to visually impaired people is collected, and the dataset is prepared manually and is used to train the deep learning model for multiple epochs. Images are augmented and manually annotated to achieve more robustness. The results demonstrate 95.19% object detection accuracy and 99.69% object recognition accuracy in real-time. The proposed work uses 0.3 s for multi-instance and multi-object detection from the captured image, which is less than a non-visually impaired person in certain scenarios. The proposed assistive system gives more information with higher accuracy in real time for visually challenged people. It can also easily differentiate between objects and obstacles coming in front of the camera.

Future work will focus on the inclusion of more objects in the dataset, which can make the dataset more efficient for the assistance of visually impaired people. More sensors will be associated with it to detect, for example, downstairs and other trajectories, giving a wider range of assistance to the visually impaired.

## Figures and Tables

**Table 1:** Number of images in each class of the collected dataset.

Total Images in Each Object Class	Original Dataset		After Augmentation		Test Set
	Training Set	Validation Set	Training Set	Validation Set	
650	350	150	3500	1500	150

**Table 2:** Performance analysis of proposed model on most relevant objects.

Objects	Total Testing Images	Correctly Detected	Detection Accuracy (%)	Correctly Recognized	Recognition Accuracy (%)
Person	150	148	98.67	148	100.00
Car	150	146	97.33	145	99.32
Bus	150	144	96.00	144	100.00
Truck	150	143	95.33	141	98.60
Chair	150	147	98.00	146	99.32
TV	150	140	93.33	140	100.00
Bottle	150	148	98.67	148	100.00
Dog	150	145	96.67	144	99.31
Fire hydrant	150	146	97.33	146	100.00
Stop Sign	150	149	99.33	147	98.66
Socket	150	143	95.33	143	100.00
Pothole	150	129	86.00	128	99.22
Pharmacy	150	141	94.00	139	98.58
Stairs	150	139	92.67	139	100.00
Washroom	150	145	96.67	145	100.00
Wrist Watch	150	140	93.33	139	99.29
Eye glasses	150	141	94.00	141	100.00
Cylinder	150	131	87.33	131	100.00
10 ₹ Note	150	141	94.00	141	100.00
20 ₹ Note	150	148	98.67	148	100.00
50 ₹ Note	150	143	95.33	143	100.00
100 ₹ Note	150	140	93.33	140	100.00
200 ₹ Note	150	144	96.00	144	100.00
500 ₹ Note	150	140	93.33	140	100.00
2000 ₹ Note	150	149	99.33	149	100.00
**Average**			**95.19%**		**99.69%**

**Table 3:** Testing accuracy and frame processing time for proposed and other methods.

Methods	Testing Accuracy	Frame Processing Time
AlexNet [38]	83.39	0.275 s
VGG-16 [39]	86.80	0.53 s
VGG-19 [40]	90.21	0.39 s
YOLO-v3	95.19	0.1 s

**Table 4:** Average time taken for different parameters.

Parameters	Average Time Taken (s)
Object Detection in single frame with GPU	0.1
Object Detection in single frame in single board DSP processor without GPU	0.3
Average time of Audio for name of object	0.4
Average time of Audio for count of object	0.2
Average time of Audio for name of object with count	0.6

**Table 5:** Processing time of each frame in different condition in single board computer.

Number of Object Class	Number of Instances of Each Object	Total Number of Objects in Frame	Average Time Taken for Object Detection (s)	Average Time of Audio Prompt (s)	Total Time to Process Single Frame (s)
0	0	0	0.3	0	0.3
1	1	1	0.3	0.4	0.7
1	2	2	0.3	0.6	0.9
1	5	5	0.3	0.6	0.9
2	1	2	0.3	0.4 + 0.4	1.1
2	2	4	0.3	0.6 + 0.6	1.5
3	5	15	0.3	0.6 + 0.6 + 0.6	2.1
4	1	4	0.3	0.4 + 0.4 + 0.4 + 0.4	1.9
4	5	20	0.3	0.6 + 0.6 + 0.6 + 0.6	2.7
5	1	5	0.3	0.4 + 0.4 + 0.4 + 0.4 + 0.4	2.3
5	3	15	0.3	0.6 + 0.6 + 0.6	2.1
5	5	25	0.3	0.6 + 0.6 + 0.6 + 0.6 + 0.6	3.3
5	10	50	0.3	0.6 + 0.6 + 0.6 + 0.6 + 0.6	3.3

**Table 6:** Comparison with state-of-the-art methods.

Method	Components	Dataset	Result	Coverage Area	Connection	Cost
Hoang et al. [41]	Mobile Kinect, laptop Electrode matrix, headphone and RF transmitter	Local dataset	Detect obstacle and generate audio warning	Indoor	Offline	High
Bai et al. [8]	Depth camera, glasses, CPU, headphone and ultrasonic sensor	Not included	Obstacle Recognition and audio output	Indoor	Offline	High
Yang et al. [42]	Depth Camera on Smart glass, Laptop, and headphone	ADE20, PASCAL, and COCO	Obstacle Recognition and generate clarinet sound as warning	Indoor, Outdoor	Internet Required	High
Mancini et al. [43]	Camera, PCB, and vibration motor	Not included	Obstacle recognition and vibration feedback for the direction	Outdoor	Offline	Low
Bauer et al. [44]	Camera, smartwatch, and smartphone	PASCAL VOC Dataset	Object detection with direction of object into audio output	Outdoor	Internet Required	High
Patil et al. [45]	Sensors, vibration motors,	No Dataset	Obstacle detection with audio output	Indoor, Outdoor	Offline	Low
Eckert et al. [46]	RGB-D camera and IMU sensors	PASCAL VOC dataset	Object detection with audio output	Indoor	Internet Required	High
Parikh et al. [47]	Smartphone, server, and headphone	Local dataset of 11 objects	Object detection with audio output	Outdoor	Internet Required	High
AL-Madani et al. [48]	BLE fingerprint, fuzzy logic	Not included	Localization of the person in the building	Indoor	Offline (Choice Wi-Fi or BLE)	Low
Proposed Method	RGB Camera, Distance Sensor, DSP processor, Headphone	Local dataset of highly relevant objects for VIP	Object detection, Count of objects, obstacle warnings, read text, and works in different modes	Indoor, Outdoor	Offline	Low
